# Adsorption Behavior of 3-phenoxybenzoic Acid by Lactobacillus Plantarum and Its Potential Application in Simulated Digestive Juices

**DOI:** 10.3390/ijms23105809

**Published:** 2022-05-22

**Authors:** Jianlong Li, Kaidi Hu, Lu Hu, Xiaoyan Hou, Qin Li, Aiping Liu, Shujuan Chen, Xiaolin Ao, Xinjie Hu, Li He, Huaqiao Tang, Daomei Huang, Yong Yang, Likou Zou, Shuliang Liu

**Affiliations:** 1 College of Food Science, Sichuan Agricultural University, Ya’an 625014, China; jlli999@sicau.edu.cn (J.L.); kaidi666@outlook.com (K.H.); HLmonet2020@163.com (L.H.); jlli666@foxmail.com (X.H.); 17888820100@163.com (Q.L.); aipliu@outlook.com (A.L.); chenshujuan1@163.com (S.C.); huavslin@163.com (X.A.); xinjiehu@sicau.edu.cn (X.H.); helifood@163.com (L.H.); yangyong676@163.com (Y.Y.); 2 Institute of Food Processing and Safety, Sichuan Agricultural University, Ya’an 625014, China; 3 College of Veterinary Medicine, Sichuan Agricultural University, Chengdu 611130, China; turtletang@163.com; 4 Integrated Agricultural Development Research Institute, Guizhou Academy of Agricultural Sciences, Guiyang 550006, China; huangdaomei915@163.com; 5 College of Resources, Sichuan Agricultural University, Chengdu 611130, China; zoulikou@sicau.edu.cn

**Keywords:** lactic acid bacteria, probiotics, bioadsorption, simulated digestive

## Abstract

3-PBA is a major degradation intermediate of pyrethroids. Its widespread existence in the environment poses a severe threat to the ecosystem and human health. This study evaluated the adsorption capacity of *L. plantarum* RS20 toward 3-PBA. Batch adsorption experiments indicated that the optimal adsorption conditions were a temperature of 37 °C and initial pH of 6.0–8.0, under which the removal rate was positively correlated with the cell concentration. In addition, there was no link between the incubation time and adsorption rate. The kinetic study showed that the adsorption process fitted well with the pseudo-second-order model, and the adsorption isotherms could be described by both Langmuir and Freundlich equations. Heat and acid treatments showed that the ability of strain RS20 in removing 3-PBA was independent of microbial vitality. Indeed, it was involved with chemisorption and physisorption via the cell walls. The cell walls made the highest contribution to 3-PBA removal, according to the adsorption experiments using different cellular components. This finding was further reconfirmed by SEM. FTIR spectroscopy analysis indicated that carboxyl, hydroxyl, amino groups, and –C–N were the functional sites for the binding of 3-PBA. The co-culture experiments showed that the adsorption of strain RS20 enhanced the degradation of 3-PBA by strain SC-1. Strain RS20 could also survive and effectively remove 3-PBA in simulated digestive juices. Collectively, strain RS20 could be employed as a biological detoxification agent for humans and animals by eliminating 3-PBA from foods, feeds, and the digestive tract in the future.

## 1. Introduction

Due to the growth of the world’s population, the pressure on food production has increased, which has led to an increase in the use of pesticides. Synthetic pyrethroid insecticides (SPs) are widely used to prevent and remove insects both in agriculture and households because of their broad-spectrum insecticidal and high effectiveness [1]. However, due to their high recalcitrance and strong hydrophobicity, SPs may eventually pose risks or threats toward animal and human life. As a key degradation intermediate of SPs, 3-phenoxybenzoic acid (3-PBA) is refractory to natural degradation, possessing a half-life of 120–180 d in soil, which is significantly longer than that of its parent compound, which is approximately 30 d [2]. Additionally, 3-PBA exhibits higher mobility and stronger polarity compared with SPs. Accumulating evidence suggests that 3-PBA can be classified as an endocrine-disrupting chemical and it demonstrates ubiquity in the environment [3,4]. In recent years, 3-PBA bioelimination using microorganisms has emerged as a research hotspot and attracted more attention [3]. Microbial methods can be mainly categorized into biodegradation and bioadsorption. Bioadsorption is considered to be a safer approach, since new toxic byproducts may be avoided. Additionally, toxic substances removal by microorganisms has outstanding advantages, including being simple to use and having low cost, high adsorption capacity, and high availability [5].

Lactic acid bacteria (LAB) are one kind of intestinal probiotic and have been employed in food fermentation for at least 4000 years, such as cheese and yogurt [6]. At present, many studies have reported that LAB can remove toxic and harmful substances, including mycotoxins, plasticizers, and heavy metals [3]. Particularly, *Lactobacillus* [7] and *Bifidobacterium* [8] are the main participants. On the other hand, LAB-derived products can also be added to poultry and livestock feedstuffs to bind mycotoxins. For instance, deoxynivalenol, fumonisins B_1_, and fumonisins B_2_ were considerably removed by inoculating LAB during fodder fermentation [9]. It is also worthy to mention that the mycotoxin adsorption ability depends on the microbial candidate, according to the findings of El-Nezami et al. that *Lactobacillus rhamnosus* GG and *Lactobacillus rhamnosus* LC-705 displayed different abilities in removing common fusarium toxins, trichothecenes [10]. LAB, as probiotics, are a kind of safe and edible microorganism commonly found in the natural environment. Many health benefits have been attributed to probiotic LAB, including the prevention of lactose intolerance, anti-carcinogenic effects, immunostimulatory effects, anti-obesity, and the reduction of serum cholesterol levels, among others [11]. To our best knowledge, there have been few studies reporting 3-PBA adsorption by LAB.

In the present study, *L. plantarum* RS20 was isolated from traditional Chinese home-made fermented vegetables (Sichuan paocai) and efficiently adsorbed 3-PBA. The characteristics and mechanism of 3-PBA adsorption by *L. plantarum* RS20 were explored. Additionally, its adsorption applications in intestinal gastric juice were evaluated, paving the way for food, feed, or intestinal probiotic products.

## 2. Results

### 2.1. 3-PBA Tolerance of L. plantarum RS20

**Figure 1:**
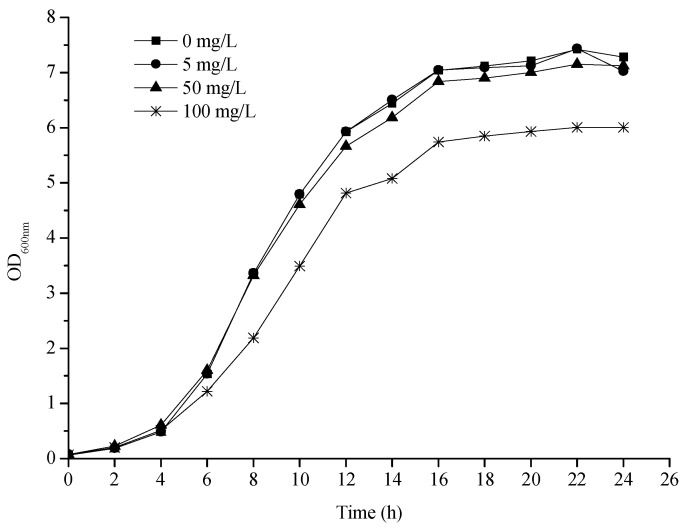
3-PBA tolerance of *L. plantarum* RS20.

Strain RS20 was isolated from Chinese home-made fermented vegetables (Sichuan paocai) and identified as *Lactobacillus plantarum* by physiological, biochemical, and molecular biology (Supplementary Figures S1–S3 and Table S1). The growth curves of *L. plantarum* RS20 at different 3-PBA concentrations are presented as Figure 1. It can be observed that the growth patterns were essentially similar in the absence and presence of 3-PBA. The strain entered the logarithmic phase after about 4 h, which was reflected in the medium’s turbidity increasing rapidly. Then, a stable phase was reached at 16 h. However, 3-PBA exerted an adverse effect on bacteria to an extent when it was at a high concentration (100 mg/L), which was embodied as the growth rate significantly slowing down. This inhibition could be ignored when the concentrations of 3-PBA were 5 and 50 mg/L, indicating that *L. plantarum* RS20 demonstrated good tolerance to 3-PBA.

### 2.2. Effects of Different Factors on 3-PBA Removal

**Figure 2:**
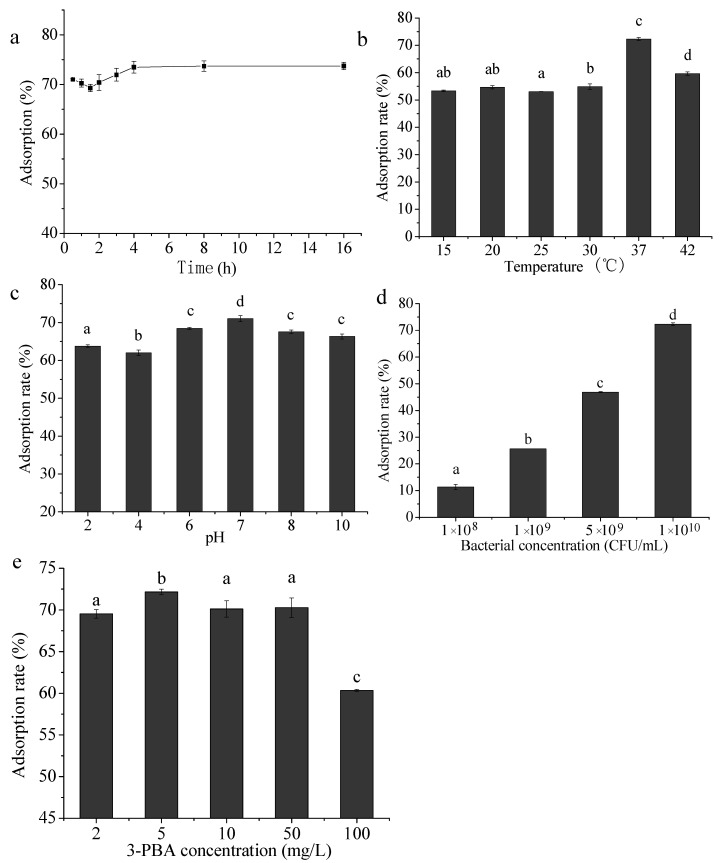
Effects of incubation time (**a**), temperature (**b**), pH (**c**), RS20 concentration (**d**), and 3-PBA concentration (**e**) on the removal of 3-PBA. Bars with different letters are significantly different (*p* < 0.05).

The 3-PBA adsorption rate of *L. plantarum* RS20 reached 71.09% since the incubation started at 0.5 h. With the extension of time, the adsorption firstly decreased to a certain extent, which may be related to the desorption behavior. After that, the adsorption rate increased slowly and gradually reached equilibrium (73.21%) at 8 h (Figure 2a). This demonstration is similar to the report of Tang et al. [12], where complete aflatoxin B_1_ adsorption was attained within 30 min by *Lactobacillus* F22. However, the adsorption efficiency decreased dramatically after 4 h, indicating that the mycotoxin adsorption by lactic acid bacteria was rapid and reversible.

As shown in Figure 2b, the 3-PBA adsorption rate changed slightly in the range of 15–30 °C and was maintained below 60%. The adsorption rate reached the highest when the temperature was 37 °C; however, it dropped below 60% again when the temperature rose to 42 °C. The same performance was observed by Khanafari et al. [13] during aflatoxin adsorption by *Lactobacillus plantarum*. In their case, the highest yield was also attained at 37 °C. In addition, Zhang et al. [14] reported that the fumonisin adsorption rate of lactic acid bacteria reached the maximum at 37 °C, then it plunged beyond this temperature, which was similar to the present study. In comparison with neutral conditions, acidic and alkaline conditions exerted negative effects on 3-PBA adsorption by *L. plantarum* RS20. In particular, the interaction between strain RS20 and 3-PBA was significantly changed under pH 2.0–6.0 and the 3-PBA adsorption rate varied from 63.79% to 68.45% (Figure 2c). Fernandez [15] found that two strains of *Enterococcus* sp. could adsorb aflatoxin efficiently when the pH was 7.0. The results are consistent with this paper, indicating that hydrogen bonding and ionic exchange may be the main interaction forces binding the cell and 3-PBA.

The effects of biomass amount and 3-PBA concentration on 3-PBA removal are presented in Figure 2d,e. As it can be seen, the adsorption rate was positively related to the biomass amount, and the effect was obvious. When the volume of the reaction system was constant, the number of bacteria increased with the increase in the concentration, and the concentration of 3-PBA remained unchanged. Similarly, the higher the biomass, the higher the adsorption of 3-PBA. With respect to the substrate concentration, the highest adsorption rate was achieved when 3-PBA was fortified at 5 mg/L. In this scenario, the adsorption rate did not greatly increase with the increase in the contaminant concentration, which may be due to the fact that there were far more adsorption sites than adsorbate. Zhang et al. [16] analyzed the adsorption capacity of *Saccharomyces cerevisiae* toward cypermethrin, and the results indicated that adsorption rate was closely related to the adsorption sites, instead of the substrate concentration.

To sum up, the best demonstration could be attained by setting the conditions as follows: pH 6.0–8.0, incubation time of 8 h, and incubation temperature of 37 °C. Additionally, there was a positive correlation between the adsorption rate and cell concentration.

### 2.3. Adsorption Kinetics

**Figure 3:**
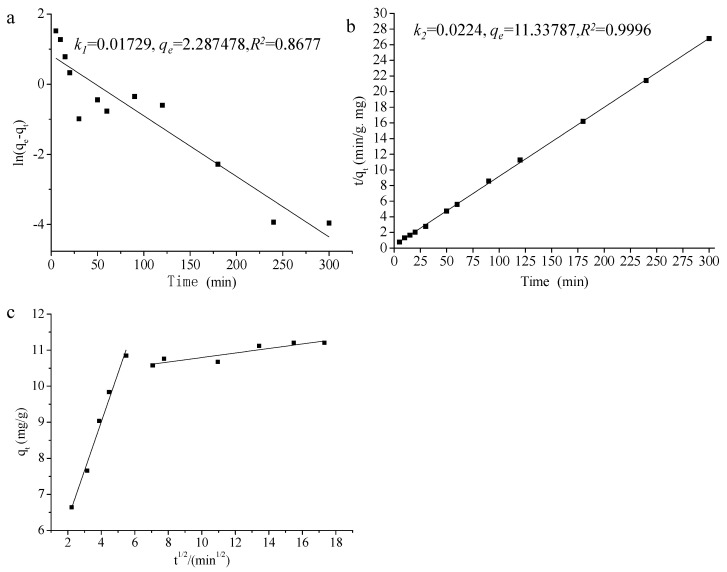
Pseudo-first/second-order kinetics equation fitting curve and Weber–Morris model for 3-PBA adsorption by *L. plantarum* RS20. Note: (**a**) is the pseudo-first-order kinetics equation; (**b**) is the pseudo-second-order kinetics equation; and (**c**) is the Weber–Morris model.

Kinetics models (pseudo-first-order, pseudo-second-order, and Weber–Morris intraparticle diffusion models) are usually used to depict the adsorption process and mechanism for traditional adsorbents [17,18]. The pseudo-first-order model and pseudo-second-order model are generally used to simulate the initial phase and all phases of biosorption [19], respectively. Figure 3a,b shows the pseudo-first/second-order model results of 3-PBA adsorption by *L. plantarum* RS20, among which the latter model showed a higher degree of fitting (R^2^ = 0.9996). 3-PBA adsorption by *L. plantarum* RS20 conformed to the pseudo-second-order model, and the equation could reflect more comprehensive and truer behaviors than the pseudo-first-order model. Therefore, it can be speculated that chemisorption is involved in 3-PBA biosorption and may be the rate-limiting step [20]. This is in line with the report of Zhang et al. [16] that cypermethrin adsorption by *Saccharomyces cerevisiae* followed the pseudo-second-order model, and similar results were also observed by Gohari [21].

The Weber–Morris model is presented in Figure 3c. The Weber–Morris model is commonly used for control analysis. The model assumes that the liquid film diffusion resistance can be ignored and the intra-particle diffusion rate constant of the adsorbent does not change along with the adsorption time and location. The adsorption process was split into two regions, and neither q_t_ nor t^1/2^ passed through the origin, indicating that intra particle diffusion was not the only step controlling the adsorption process. The initial step was a rapid reaction, in which 3-PBA molecules were diffused from the solution to the bacterial surface through the liquid membrane in a short time. Then, adsorption approached equilibrium after about 30 min (t^1/2^ = 5.48), and the combination of 3-PBA with the cellular surface’s active functional groups occurred in this second step [22].

### 2.4. Adsorption Isotherms

Several mathematical models are used to evaluate the adsorption strength and predict the biosorption capacity [23]. The Langmuir and Freundlich isotherm models were used to analyze the data regarding 3-PBA biosorption in the present study. The calculated parameters are given in Table 1—the Langmuir model was more suitable than the Freundlich model, with a higher R^2^ value (0.989). Similarly, Dai et al. [24] reported that the adsorption of Pb (II) by *Lactobacillus brevis* can be well fitted by Langmuir isotherm models. From the parameters of the Langmuir isotherm, the equilibrium adsorption value reached 10.725 mg/g, which indicates that the adsorption process seemed to involve physisorption.

### 2.5. Adsorption Mechanism of 3-PBA

#### 2.5.1. Effects of Pretreatment on the 3-PBA Adsorption Ability

**Figure 4:**
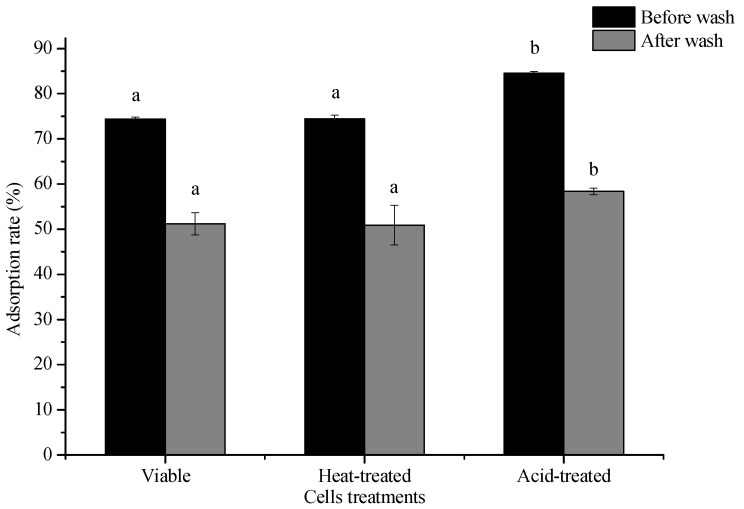
Influence of heat-inactivated and acid-inactivated *L. plantarum* RS20 cells on the 3-PBA-removing capacity. Note: different lower case letters between different treatments mean significant differences (*p* < 0.05).

Cells were deactivated using heat and acid. Their 3-PBA adsorption capacity was investigated and compared to that of live cells. The results are shown in Figure 4. The adsorption ability of *L. plantarum* RS20 toward 3-PBA did not decline after different treatments. Instead, higher removals (*p* < 0.05) were obtained using heating and acid in comparison with the control, with adsorption rates of 74.44% and 84.53%, respectively.

Studies have shown that surface adsorption by lactic acid bacteria is related to peptidoglycans and polysaccharides, which are extremely sensitive to heat and acid treatment. Heat denatures proteins and causes a Maillard reaction between polysaccharides and proteins, whereas acid treatment destroys the glycosidic bond of polysaccharides, that is, it destroys the peptidoglycan structure, which could probably explain the rise in the 3-PBA adsorption capacity after acid treatment (Figure 4). Wang et al. [25] found that the main factors affecting penicillin absorption by *Lactobacillus brevis* 20,023 were polysaccharides and proteins, mainly concerning the functional groups C–O, OH, and/or NH.

Figure 4 shows that the surface adsorption by lactic acid bacteria was not significantly different between the viable and heat-treated bacteria, indicating that the adsorption process was not dependent on biomass viability. This phenomenon can be ascribed to the adsorption process not being biodegradation or biotransformation, but biosorption [26]. Similar results were reported by McKinley et al. [27], where aflatoxin B_1_ and zearalenone adsorption by gastro-intestinal flora were investigated. It was found that the inactivated strains exhibited the same or even higher effectiveness in addressing these toxins compared with live strains, which is consistent with the results obtained in present study, showing that the 3-PBA adsorption rate significantly decreased before and after heat treatment. On the contrary, Hatab et al. [28] reported that there was no significant difference in the patpenicillin removal ability of dead lactic acid bacteria and live ones. The reason is that mycotoxin removal by *Bifidobacterium bifidum* 6071 and *Lactobacillus rhamnosus* 6149 mainly depended on adsorption to the cellular surface.

Nowadays, numerous biological technologies have been developed to remove toxic substances from the environment, which have shed light on on-site remediation. However, choosing a suitable biomaterial is a major challenge in this context. At present, living/dead microorganisms and surface-modified biomass are the most prevailing candidates. With respect to *L. plantarum* RS20, the adsorption process was not dependent on biomass viability. Extensive literature suggests that the probiotic nature of LAB depends on peptidoglycan, polysaccharides, or metabolites. Therefore, *L. Plantarum* RS20 can be used as a biomaterial to remove 3-PBA, and probiotics can be kept in the intestines when used in feed or food in the future.

Figure 4 also shows that the adsorption rates in all treatment groups decreased (23%) after eluting with PBS buffer solution. This was the consequence of desorption, which indicated that non-covalent bonding (physical adsorption) existed during 3-PBA adsorption. This observation is consistent with the effect of pH on the adsorption rate, since the adsorption rate of 3-PBA significantly changed under pH 2.0–6.0, indicating that hydrogen bonding and ionic exchange may have been the main interaction forces binding the cell and 3-PBA. Similar results were reported previously by Shen et al. [29], where acrylamide was physically adsorbed by *L. plantarum*, which was closely associated with the cell wall roughness, hydrophobicity, nitrogen-to-carbon (N/C) ratio, and functional groups.

#### 2.5.2. Adsorption Ability of Different Cellular Structures

**Figure 5:**
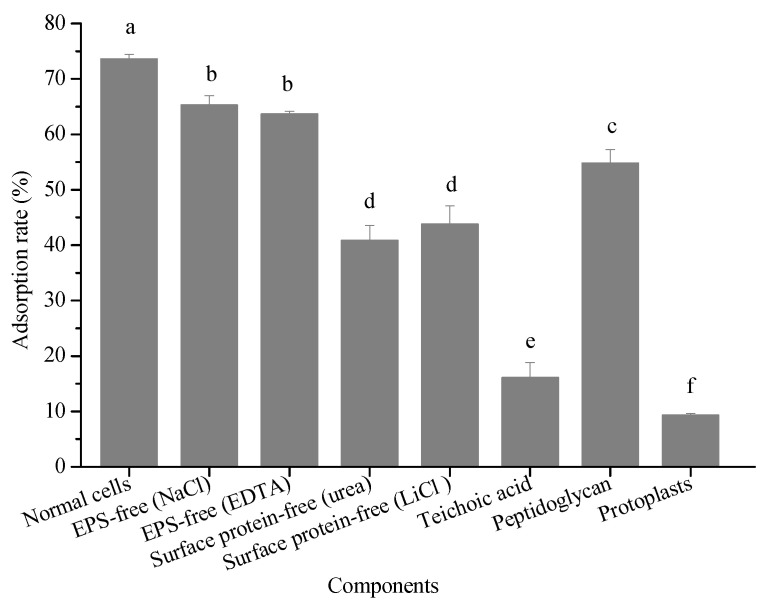
Adsorption abilities of different cellular components. Note: parentheses mean that different methods were used to remove EPS or surface proteins. Bars with different letters are significantly different (*p* < 0.05).

To our best knowledge, most researchers agree with the mechanism that proteins, carbohydrates, peptidoglycan, polysaccharides, and other substances in the lactic acid bacterial cell wall play key roles within the pollutant adsorption process [30], so this aspect was also considered in the present study. Figure 5 shows the adsorption ability of bacterial cells in the absence of either EPS or surface proteins, and the cell wall toward 3-PBA. A significant difference in terms of the adsorption rate was observed between the cells in the absence and presence of EPS, which indicates that EPS contributed to 3-PBA adsorption. The cell walls exhibited the greatest capability to remove 3-PBA (70.92%). Additionally, the 3-PBA adsorption rate of the bacterial cells in the absence of the cell surface protein was 40.86–43.79%. In contrast, the adsorption rate was only 9.32% for the protoplast. These results suggest that there are many adsorption sites on cell walls, among which the cell surface protein plays a significant role. Haskard et al. investigated the adsorption mechanism of aflatoxin B_1_ by *L. rhamnosus* strain GG and *L. rhamnosus* strain LC-705 and found that the extracellular bacterial surface affected the process [31].

*L. plantarum* RS20 is a Gram-positive bacterium, the main components of the cell wall of which are peptidoglycan and teichoic acid. Their 3-PBA adsorption rates are 54.83% and 16.09%, respectively. Therefore, peptidoglycan played the main role in 3-PBA adsorption in the cell wall. However, different findings were reported by Zhang et al. [32] in the case of patulin adsorption by yeast, where physical adsorption was mainly due to proteins and polysaccharides. Hence, further research is needed to address the mechanisms behind this.

#### 2.5.3. SEM Analysis

**Figure 6:**
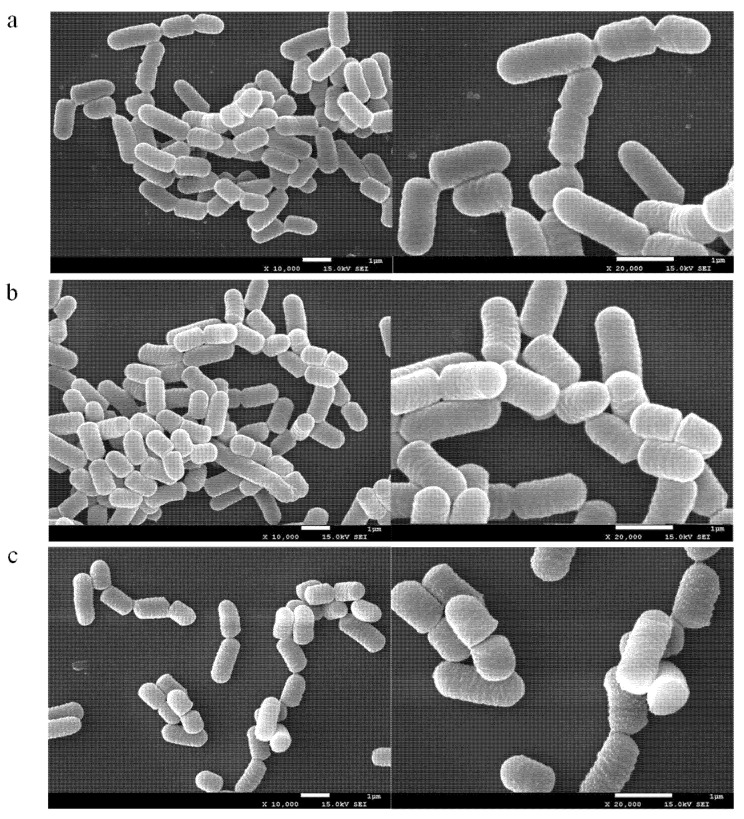
SEM photographs of *L. plantarum* RS20 biomass under three different 3-PBA concentrations ((**a**) control group, (**b**) 5 mg/L, and (**c**) 100 mg/L).

The effect of 3-PBA on the cells was analyzed by SEM. The contaminant was spiked at two different concentrations, 5 mg/L and 100 mg/L. As shown in Figure 6, compared with the blank control, cells retained their short rod structure and their appearance did not change significantly after adsorption when 3-PBA was fortified at 5 mg/L. However, the cell surface became rough, together with distinct granular material adhering to the surface after raising the substrate concentration to 100 mg/L 3-PBA. On the other hand, none of the cells in the experimental group exhibited cleavage and perforation. Therefore, based on the above results, it can be inferred that 3-PBA binds to the active sites on the cell’s surface.

#### 2.5.4. FT-IR Analysis

**Figure 7:**
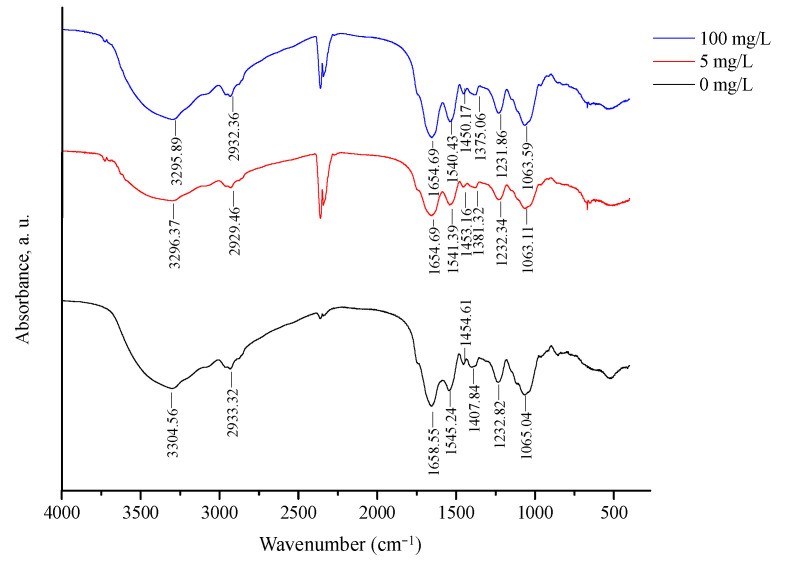
FTIR spectra of *L. plantarum* RS20 powder biomass.

To better understand the surface adsorption mechanism, FT-IR analysis was employed to identify potential functional groups participating in 3-PBA removal by *L. plantarum* RS20 (Figure 7). Characteristic peaks were identified according to the references [33,34]. Compared with the FT-IR spectra of the control, the peak position of the bound 3-PBA shifted. The peak at 3304 cm^−1^ was the result of –OH and N–H stretching vibrations. The wavenumber shifted to 3296 and 3295 cm^−1^, respectively, after exposure to 3-PBA at 5 and 100 mg/L, indicating that –OH/–N–H contributed to 3-PBA adsorption. The peaks at 2933, 1658, 1545, 1454, and 1065 cm^−1^ generally shifted toward lower wavenumbers when 3-PBA was present. The absorption peak at 1445 cm^−1^ was in the amide II region, and it corresponded to a combination of the N–H in-plane bending and the –CN stretching vibration [35]. The peaks at 1407 cm^−1^ shifted to 1381 cm^−1^ and 1375 cm^−1^, which were assigned to amide III, indicating that amide promoted 3-PBA adsorption. The observed changes indicated that the –OH, –N–H, –C–N, and –C–O groups were involved in the 3-PBA biosorption process. These functional groups have also been reported to be involved in other xenobiotics’ bio adsorption, including cypermethrin, patulin, and Cd^2+^ [16,28,36].

### 2.6. 3-PBA Adsorption Assay under Simulated GI Conditions

**Figure 8:**
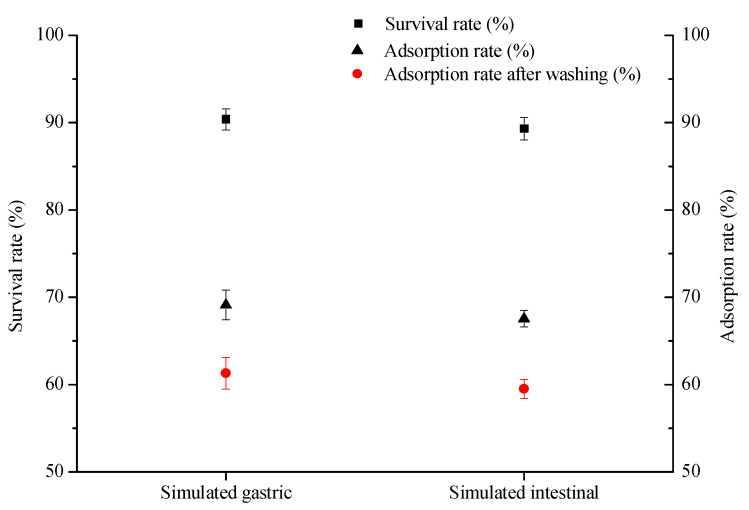
Tolerance of *L. plantarum* RS20 to simulated gastric and simulated intestinal juices.

Considering that the application of lactic acid bacteria as probiotics in food and feed has great potential [37,38], we evaluated the tolerance of *L. plantarum* RS20 to the gastrointestinal tract via simulated gastric and intestinal juices. As shown in Figure 8, it can be seen that the survival rates of *L. plantarum* RS20 were more than 90% in simulated gastric and intestinal juices after treatment, indicating that this strain could tolerate the simulated GI tract conditions and remain relatively viable. Zoghi et al. reported that *L. plantarum* has a high ability to remove patulin under GI conditions [39]. In comparison with intestinal juice, the 3-PBA adsorption ability was higher after gastric juice treatment, which is consistent with the previous results that acid treatment could enhance the adsorption capacity (Section 2.5.1). Meanwhile, a low quantity of 3-PBA was released after washing, which suggests that strain RS20 can safely remove 3-PBA when added to food or feed. These findings are in agreement with the study of Zhao et al., where *Leuconostoc mesenteroides* DM12 exhibited a high adsorption rate in a simulated gastrointestinal system [40]. Therefore, *L. plantarum* RS20 can survive and remove 3-PBA in simulated digestive juices, indicating that this strain is a promising adsorbent candidate for application in vivo.

### 2.7. Degradation of 3-PBA by Cooperation of RS20 and SC-1

**Figure 9:**
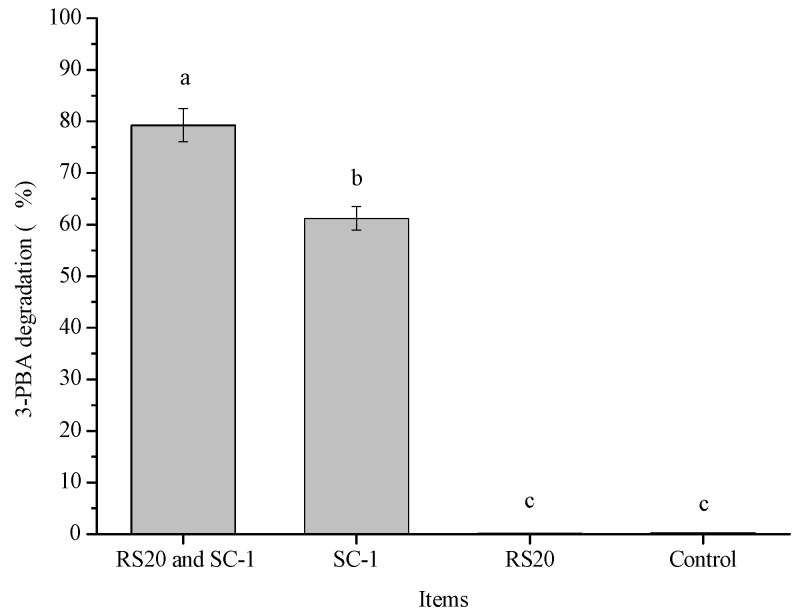
Degradation of 3-PBA by the cooperation of strain RS20 and strain SC-1. Note: Bars with different letters are significantly different (*p* < 0.05).

Figure 9 shows the degradation of 3-PBA by the cooperation of RS20 and SC-1. The degradation of 3-PBA in co-culture was significantly higher than that of the experimental group inoculated with SC-1 alone (79.23% and 61.21%, respectively). The possible reason for this was that 3-PBA was enriched by RS20 adsorption, which was conducive to the degradation of 3-PBA by SC-1. The results showed that the complex bacterial system of RS20 and SC-1 had great application potential in the elimination of 3-PBA in food and the environment.

Kaolinite has strong adsorption capacity for phenol, and it was found to be the most effective in accelerating the degradation rate, as well as improving the degradation efficiency by *Sphingomonas* sp. GY2B [41]. Similar results can be found in this paper. The adsorption of RS20 on 3-PBA could provide protection for SC-1 and partially resist the adverse effects of substrate inhibition. Liu et al. [42] studied the degradation of cypermethrin and its metabolites (3-PBA) by a co-culture of *Bacillus licheniformis* B-1 and *Sphingomonas* sp. SC-1. The degradation of 3-PBA by SC-1 promoted the positive metabolism of cypermethrin and accelerated the degradation of cypermethrin by B-1. Therefore, co-culture technology has certain application potential in the elimination of toxic substances.

## 3. Experimental Design

### 3.1. Microorganism

*Lactobacillus plantarum* RS20 was obtained from Sichuan paocai (Meishan, China). *Sphingomonas* SC-1 that could degrade 3-PBA completely was isolated from activated sludge and was proven to be safe by a bacterial toxicological experiment. *Lactobacillus plantarum* RS20 and *Sphingomonas* SC-1 were preserved in the microbiological laboratory of the College of Food Science, Sichuan Agricultural University.

### 3.2. Chemicals and Reagents

3-Phenoxybenzoic acid (3-PBA, 98% purity) was purchased from Sigma-Aldrich Chemical Co. (Shanghai, China). Chromatographic-grade acetonitrile was obtained from CNW Technologies GmbH (Dusseldorf, Germany). All other chemicals were of analytical grade.

A 3-PBA working solution was prepared by dissolving an appropriate quantity of the substance in 1 mL of methanol, followed by adding Milli-Q water up to 10 mL. Then, the solution was subsequently filtered through a 0.22 μm membrane and stored at 4 °C until use.

### 3.3. Biomass Preparation

*L. plantarum* RS20 was cultured in MRS broth (De Man, Rogosa, Sharpe, Difco^TM^) at 30 ± 1 °C for 20 h, thereby achieving a cell concentration of about 1.0 × 10^10^ cfu/mL. The culture was employed as the fermentation component (FC). Then, after centrifuging at 7.24 × 10^3^× *g* (4 °C) for 10 min, the precipitate was washed three times with phosphate buffer solution (PBS, 0.02 M, pH 7.2). The obtained pellet was considered as the active cell component (AC), from which the freeze-dried component (FDC) was prepared and stored at −20 °C.

### 3.4. Stress of 3-PBA on L. plantarum RS20

*L. plantarum* RS20 was cultured in MRS broth using 96-well plates at 37 °C for 24 h in the absence and presence of 3-PBA at different concentrations (0, 5, 50, and 100 mg/L). The OD value at 600 nm was measured using an enzyme-labeled instrument (Varioskan LUX Multimode, Thermo Fisher Scientific, Waltham, MA, USA).

### 3.5. Batch Experiments

Batch adsorption experiments were conducted in 10 mL of Eppendorf, where the reaction system consisted of 5 mL of bacterial suspension and 3-PBA working solution. Factors including the incubation time (0.5, 1, 2, 4, 6, 8, and 12 h), temperature (15 °C, 20 °C, 25 °C, 30 °C, 37 °C, and 42 °C), pH (2.0, 4.0, 6.0, 7.0, 8.0, and 10.0), 3-PBA concentration (2, 5, 10, 50, and 100 mg/L), and biomass amount (1.0 × 10^8^, 1.0 × 10^9^, 5.0 × 10^9^, and 1.0 × 10^10^ cfu/mL) were selected as the independent variables. The control was prepared under the same conditions in the absence of *L. plantarum* RS20. Cultures were incubated on a rotary shaker at 55.9× *g*, unless otherwise mentioned. After the designed period, the supernatant was obtained through centrifugation (2.9 × 10^4^× *g*, 10 min, 4 °C) and subjected to 3-PBA residue determination.

### 3.6. Kinetic Study

The FDC was suspended in 0.5–50 mg/L 3-PBA working solution, and the cell concentration was adjusted to 1 g/L (*m*/*v*). The mixture was incubated at 37 °C for 5–360 min. In this case, the pseudo-first-order, pseudo-second-order, and Weber–Morris models were adopted to fit the obtained data.

### 3.7. Biosorption Isotherm

Two equilibrium isotherms, including the Langmuir and Freundlich models, were used to describe the biosorption equilibrium [43]. The experimental steps were as follows: 1 mg of FDC was inoculated with 1 mL of 3-PBA working solution (20 mg/L). The mixture was incubated for 5–300 min at 37 °C.

### 3.8. Adsorption Mechanism of 3-PBA

#### 3.8.1. Effect of Different Treatments on Adsorption Ability

Heat treatment: The AC was autoclaved at 121 °C for 15 min, followed by resuspension in 1 mL of 5 mg/L 3-PBA and incubating for 8 h at 37 °C. After centrifugation, a sample was taken from the suspension to evaluate the 3-PBA adsorption ability.

Acid treatment: The AC was resuspended in 2 M HCl for 90 min, then centrifuged at 7.24 × 10^3^× *g* for 10 min at 4 °C. The cell precipitation was washed three times with PBS (0.02 M and pH 7.2) and resuspended in 1 mL of 5 mg/L 3-PBA. The mixture was incubated for 8 h at 37 °C. Afterward, the 3-PBA residues were analyzed by HPLC.

Stability of adsorption ability: The AC treated with heating and acid were incubated in 1 mL of 3-PBA solution (5 mg/L) at 37 °C for 8 h, then centrifuged at 7.24 × 10^3^× *g* for 10 min at 4 °C. The cell precipitation was washed three times with PBS (0.02 M, pH 7.2) and resuspended in 1 mL of PBS (0.02 M, pH 7.2). After 1 h of incubation, the supernatant was collected and subjected to 3-PBA residue analysis.

#### 3.8.2. Adsorption Ability of Different Cellular Structures

##### Effect of Exopolysaccharides (EPS)

AC was suspended in 1.0 M NaCl and underwent ultrasonication for 5 min (100 W, 20–25 kHz, and 1 s interval every 4 s) in an ice bath. Then, the cell pellet was treated with 0.05 M EDTA. After 12 h of incubation with 5 mg/L 3-PBA, the sample was collected and subjected to 3-PBA residue analysis.

##### Effect of Bacterial Surface Protein

AC was treated with 5 M LiCl at 4 °C for 60 min, followed by 8 M urea solution at 37 °C for 2 h. The cell precipitation was washed three times with PBS (0.02 M, pH 7.2) and resuspended in 1 mL of 5 mg/L 3-PBA. The mixture was incubated for 8 h at 37 °C. Afterward, cells were collected to assess the 3-PBA adsorption rate.

##### Adsorption Ability of Cellular Components

AC was suspended in PBS buffer and crushed by ultrasonic disruption for 60 min (400 W, 20–25 kHz, and 1 s interval every 4 s) in an ice bath. After centrifugation, the supernatant was collected as the cell-free extract (CFE). 3-PBA was fortified in the CFE, reaching a concentration of 5 mg/L. The mixture was incubated at 37 °C for 8 h. Afterward, the 3-PBA concentration was determined using HPLC. Simultaneously, the precipitate was washed twice with sterile PBS buffer and transferred to sodium dodecyl sulfate (SDS) (8 g/L). The mixture was incubated in boiling water for 10 min to separate protein substances [44]. The SDS was removed by washing with sterile ultrapure water and resuspended in sterile PBS buffer. Then, trypsin was added to a concentration of 3 mg/mL and the matrix was further incubated at 37 °C for 12 h. After centrifugation, the precipitate was washed twice with sterile PBS buffer to obtain the cell wall (CW). The peptidoglycan and protoplasts were prepared in accordance with the methods reported by Rolain et al. [45] and Yu et al. [46]. A sample was collected to evaluate the 3-PBA adsorption ability.

#### 3.8.3. SEM Analysis

FC was resuspended in 3-PBA working solutions with 5 and 100 mg/L and incubated at 37 °C for 8 h. After centrifugation (7.24 × 10^3^× *g*, 10 min), cells were firstly fixed with 2.5% glutaraldehyde overnight at 4 °C, and then successively dehydrated in 30, 50, 70, 80, 90, and 100% ethanol. Finally, they were further dried using a critical point dryer and subsequently coated using a Sputter coater (SPT-20, Coxem, Daejeon, Korea) in order to improve the quality of the images [47]. Field-emission scanning electron microscopy (FESEM) images were collected on a JEOL JEM-7500F scanning electron microscope.

#### 3.8.4. Fourier-Transform Infrared Spectrum (FT-IR) Analysis

AC was resuspended in the 3-PBA working solution (100 mg/L) and incubated at 37 °C for 8 h. Afterward, cells were washed with PBS (0.02 M, pH 7.2) and freeze dried. An amount of 1.0 mg of cells was ground with 100 mg of anhydrous KBr powder and the spectra were recorded at 4000 to 400 cm^−1^ by a spectrophotometer (Nicolet iS5, Thermo Fisher Scientific, Waltham, MA, USA).

### 3.9. 3-PBA Adsorption Assay in Simulated Gastrointestinal Conditions

Simulated gastric and intestinal juices were prepared as described by Zhang et al. [16], where distilled water was replaced with the 3-PBA working solution, achieving a final concentration of 5 mg/L. The initial cell concentration was 1 × 10^9^ cfu/mL and the culture was placed in a shaking water bath maintained at 37 °C for 2 h. The survival rate was calculated in accordance with the equation of Guo et al. [48] (Equation (2)). Then, a sample was collected, and 3-PBA residues were analyzed by HPLC.

### 3.10. Degradation of 3-PBA by Cooperation of RS20 and SC-1

Mixed inocula of RS20 and SC-1 at 1:1 biomass ratios were prepared, and the cell densities of the mixed inocula were all 1.0 × 10^8^ cfu/mL. Each inoculum (1.5 mL) was separately inoculated to 30 mL of MRS broth with 100 mg/L 3-PBA. Uninoculated MRS broth with 100 mg/L 3-PBA served as the control. All of the inocula were cultivated using a shaker at 30 °C and 7.2× *g* for 72 h. Then, the 3-PBA contents (mg/L) in the media were measured.

### 3.11. Analytical Methods

The residual 3-PBA concentration was analyzed using high-performance liquid chromatography (HPLC, LC-2010, Shimadzu, Japan) equipped with a C18 column (InertSustain, 250 mm × 4.6 mm, 5.0 μm). Acetonitrile (A) and phosphoric acid water (B, pH 2.5) were used as the mobile phase. An isocratic elution was performed by employing A:B = 55:45 at a flow rate of 1 mL/min. The injection volume was 20 μL. The detection wavelength was set as 210 nm. The 3-PBA adsorption rate (%) was calculated using Equation (1).

(1) $$\mathrm{Adsorption}\;\mathrm{rate}\;(\%)=[1\;-\;\frac{S\;}{C\;}]\;\times \;100\%$$

where S and C correspond to the 3-PBA concentration in the experimental supernatant and control, respectively.

The survival rate was calculated according to the following equation:

(2) $$\mathrm{Survival}\;\mathrm{rate}\;(\%)=\frac{{\mathrm{logcfu}\;N}_{1}\;}{{\mathrm{logcfu}\;N}_{0}\;}\;\times \;100\%$$

where N_1_ is the total viable count of strain RS20 after treatment with distilled water or simulated gastrointestinal juices. N_0_ represents the total viable count of strain RS20 before treatment.

The pseudo-first-order model of adsorption kinetics could be expressed as follows:

(3) $$\ln\;\left(q_{e}-q_{t}\right)={\;\ln\;q}_{e}-K_{1}t$$

where q_e_ is the 3-PBA biosorption equilibrium capacity (mg/g), q_t_ is the amount of 3-PBA adsorbed at any time t, and K_1_ corresponds to the rate constant of pseudo-first-order kinetics (min^−1^).

The pseudo-second-order model of adsorption kinetics is presented in Equation (4):

(4) $$\frac{t}{q_{t}}=\frac{1}{q_{e}^{2}}+\frac{1}{K_{2}q_{e}}t$$

where q_t_ is the amount of 3-PBA adsorbed at time t (min), q_e_ is the equilibrium uptake of 3-PBA (mg/g), and K_2_ represents the pseudo-second-order rate constant (g/mg·min).

The intraparticle diffusion model (Weber–Morris model) can be used to analyze the adsorption kinetics, and its formula is as follows:

(5) $$q_{t}{=k}_{\mathrm{id}}t^{1/2}+C$$

where q_t_ (mg/g) is the 3-PBA uptake at time t (min), K_id_ is the intraparticle diffusion rate constant (mg/[g·min^1/2^]), and C is the constant related to the boundary layer effect of adsorption. If the adsorption process is completely controlled by intraparticle diffusion, the plot of q_t_ versus t^1/2^ passes through the origin [49].

The Langmuir isotherm model describes a monolayer adsorption process, presented as Equation (6) [50]:

(6) $$q_{e}=\frac{q_{\max}K_{L}C_{e}}{{1+K}_{L}C_{e}}$$

where C_e_ is the equilibrium 3-PBA concentration in solution (mg/L), q_e_ is the amount of 3-PBA absorbed in the equilibrium state, q_max_ is the maximum adsorption capacity (mg/g), and K_L_ corresponds to the Langmuir constant and indicates the affinity between adsorbents and adsorption sites.

The Freundlich isotherm model assumes that the adsorption heat of multilayer adsorption on a heterogeneous adsorption surface is not uniformly distributed [50]. This model is expressed as Equation (7):

q_e_ = K_F_Ce^1/n^ (7)

where C_e_ is the equilibrium 3-PBA concentration in solution (mg/L), q_e_ is the amount of 3-PBA absorbed in the equilibrium state, and K_F_ and n are the Freundlich constants representing the adsorption capacity and adsorption intensity, respectively.

All the tests were carried out at least in triplicate. Data analysis was performed using the software of SPSS (version V22.0, IBM SPSS, USA Almonk). The value of *p* < 0.05 was considered statistically significant and analyses were conducted using ANOVA using the SPSS V22.0 software.

## 4. Conclusions

3-PBA adsorption by *L. plantarum* RS20 was strongly affected by the temperature, pH, cell concentration, and 3-PBA concentration. In particular, the incubation time exerted almost no effect on the adsorption rate, while it was positively correlated with the cell concentration. The kinetic study showed that the biosorption kinetic data fitted the pseudo-second-order kinetic model, and the Langmuir and Freundlich equations were more suitable for the data of the adsorption of 3-PBA by *L. plantarum* RS20. The biomass viability was not the prerequisite for 3-PBA adsorption, which mainly occurred on the cell surface. The results of the adsorption test using different cellular structures indicated that the adsorption process involved chemisorption and physisorption, during which the cell walls and the protoplasts played a major role in 3-PBA adsorption. The FTIR spectra showed numerous functional groups in *L. plantarum* RS20 that could easily bind 3-PBA. In particular, the co-culture system of RS20 and SC-1 could improve the degradation of 3-PBA, indicating that *L. plantarum* RS20 displayed great potential in removing 3-PBA from food, feed, and the environment.

## Figures and Tables

**Table 1:** Adsorption constants derived from simulations with different isotherm models.

Isotherm Parameters	K	q_max_ (mg/g)	1/n	R^2^
Langmuir	0.0539	10.7250	—	0.9890
Freundlich	1.2560	—	0.4480	0.9340

## Supplementary Materials

The following supporting information can be downloaded at: www.mdpi.com/article/10.3390/ijms23105809/s1.
