# Supplementary material for: Adsorption Behavior of 3-phenoxybenzoic Acid by Lactobacillus Plantarum and Its Potential Application in Simulated Digestive Juices
Source: Int J Mol Sci. 2022 May 22;23(10):5809. doi: 10.3390/ijms23105809 (PMC9146835; doi:10.3390/ijms23105809)
Supplement: Supplementary file 1 [file ijms-23-05809-s001.zip › ijms-1716745-Supplementary figure.pdf]

Supplementary materials

Figure S1 3-PBA-removal-capacity of lactic acid bacteria strains

Figure S2 Cell morphology and colony morphology of strain RS20

Figure S3 Phylogenetic tree of strain RS20

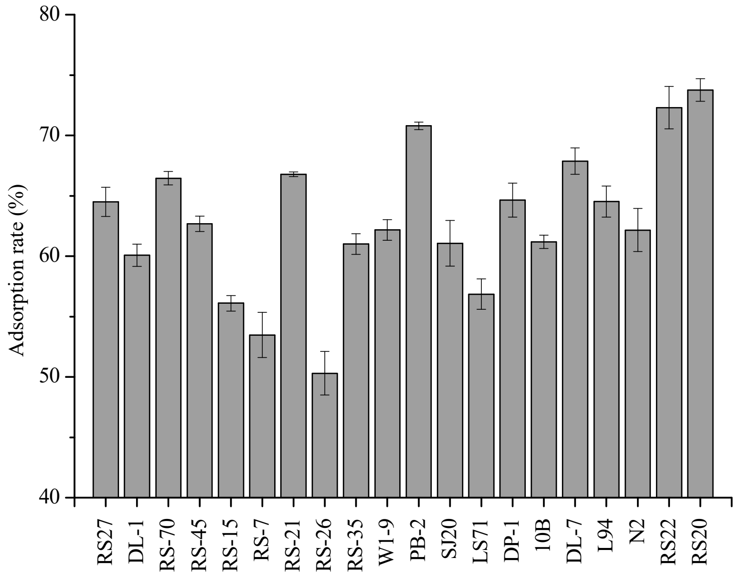

Figure S1

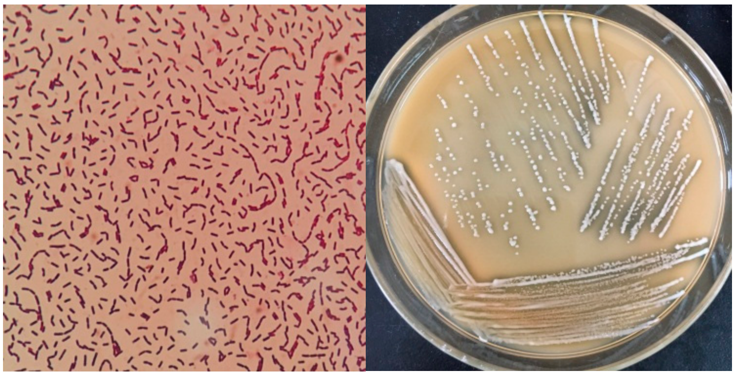

Figure S2

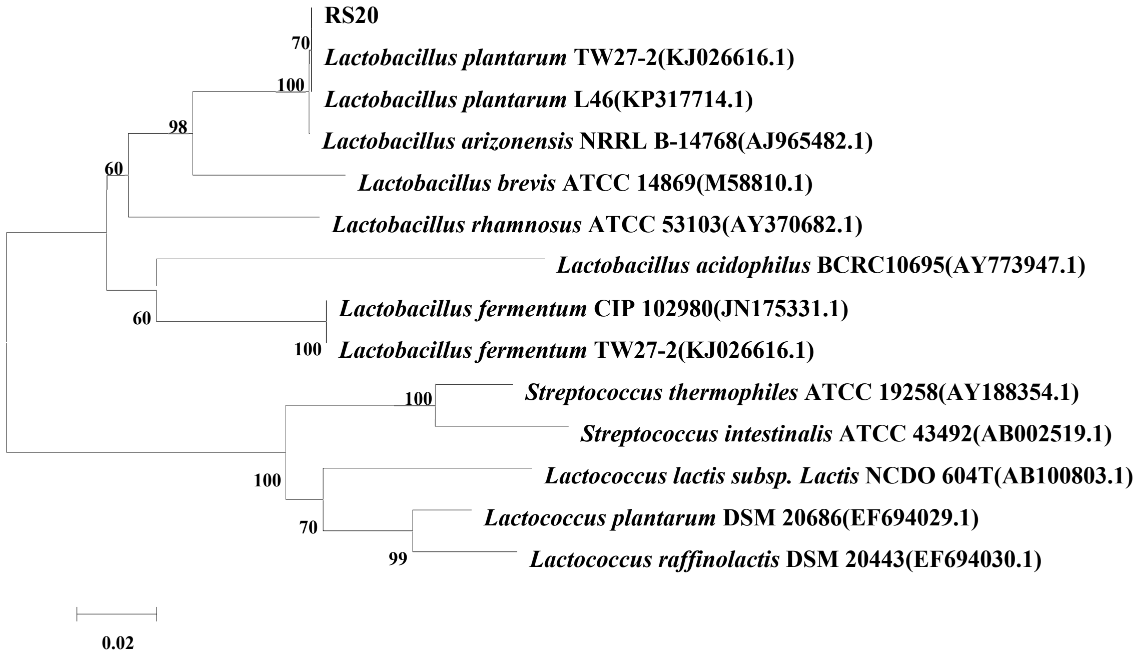
RS20
70
Lactobacillus plantarum TW27-2(KJ026616.1)
100
Lactobacillus plantarum L46(KP317714.1)
98
Lactobacillus arizonensis NRRL B-14768(AJ965482.1)
60
Lactobacillus brevis ATCC 14869(M58810.1)
Lactobacillus rhamnosus ATCC 53103(AY370682.1)
Lactobacillus acidophilus BCRC10695(AY773947.1)
60
Lactobacillus fermentum CIP 102980(JN175331.1)
100
Lactobacillus fermentum TW27-2(KJ026616.1)
100
Streptococcus thermophiles ATCC 19258(AY188354.1)
Streptococcus intestinalis ATCC 43492(AB002519.1)
100
Lactococcus lactis subsp. Lactis NCDO 604T(AB100803.1)
70
Lactococcus plantarum DSM 20686(EF694029.1)
99
Lactococcus raffinolactis DSM 20443(EF694030.1)
0.02

Figure S3
