# Supplementary material for: Adsorption Behavior of 3-phenoxybenzoic Acid by Lactobacillus Plantarum and Its Potential Application in Simulated Digestive Juices
Source: Int J Mol Sci. 2022 May 22;23(10):5809. doi: 10.3390/ijms23105809 (PMC9146835; doi:10.3390/ijms23105809)
Supplement: Supplementary file 1 [file ijms-23-05809-s001.zip › ijms-1716745-Supplementary table.pdf]

Supplementary materials

Table S1 Physiological and biochemical characteristics of strain RS20

Table S1

Items	RS20	Items	RS20
Contact enzyme	-	Lactose	+
Oxidase enzyme	-	Maltose	+
Gelatin Liquefaction	-	Mannose	+
Nitrate reduction	-	Cottonseed sugar	+
Indole	-	Salicin	+
Hydrogen sulfate	-	Sorbitol	+
Arginine produces ammonia	-	Sucrose	+
Cellobiose	+	15℃	+
Esculin hydrate	+		
